# Diabetes-related research priorities of people with type 1 and type 2 diabetes: a cross-sectional study in Germany

**DOI:** 10.1038/s41598-022-24180-y

**Published:** 2022-12-02

**Authors:** Sandra Olivia Borgmann, Marlo Verket, Veronika Gontscharuk, Bettina Bücker, Sabine Arnolds, Olaf Spörkel, Stefan Wilm, Andrea Icks

**Affiliations:** 1 Institute for Health Services Research and Health Economics, German Diabetes Center (DDZ), Leibniz Center for Diabetes Research at the Heinrich Heine University Düsseldorf, Auf’m Hennekamp 65, 40225 Düsseldorf, Germany; 2 Institute for Health Services Research and Health Economics, Centre for Health and Society, Medical Faculty and University Hospital Düsseldorf, Heinrich Heine University Düsseldorf, Moorenstr. 5, 40225 Düsseldorf, Germany; 3 German Center for Diabetes Research (DZD), Partner Düsseldorf, Ingolstädter Landstraße 1, 85764 München-Neuherberg, Germany; 4 National Diabetes Information Center, German Diabetes Center (DDZ), Leibniz Center for Diabetes Research at the Heinrich Heine University Düsseldorf, Auf’m Hennekamp 65, 40225 Düsseldorf, Germany; 5 Institute of General Practice, Centre for Health and Society, Medical Faculty and University Hospital Düsseldorf, Heinrich Heine University Düsseldorf, Moorenstr. 5, 40225 Düsseldorf, Germany; 6 Profil Institut für Stoffwechselforschung GmbH, Hellersbergstraße 9, 41460 Neuss, Germany; 7 Present Address: Department of Internal Medicine I, University Hospital Aachen, RWTH Aachen University, Aachen, Germany

**Keywords:** Diabetes, Health care

## Abstract

To investigate (i) the importance and priorities of research objectives for people with type 1 (T1DM) and type 2 diabetes (T2DM); (ii) subgroups with specific research priorities; (iii) associated factors (e.g., sociodemographic characteristics) of the subgroups. The cross-sectional survey was conducted in 2018 using data from 869 respondents (29.0% response, 31.2% female, mean age 61.3 years, 62.7% T2DM) from a German statutory health insurance population. Diabetes-related research priorities were assessed with a questionnaire. Subgroups and associated factors were identified using latent class analysis. Three subgroups were found in T1DM: (1) high priority for the research topic ‘healing diabetes’ and moderate priority for the research topic ‘prevention of long-term complications’, (2) priorities for simplifying handling (high) and stress reduction (moderate), (3) priorities for healing diabetes (high) and simplifying handling (high). Three subgroups were found in T2DM: (1) priorities for simplifying handling (moderate), diabetes prevention (moderate) and prevention of long-term complications (moderate), (2) priorities for stress reduction (high) and diabetes prevention (moderate), (3) priorities for simplifying handling (high) and stress reduction (high). Classes differed in age and HbA_1c_. Knowledge about research priorities enables researchers to align their work with the needs of people with diabetes.

## Introduction

Diabetes mellitus (DM) is a chronic health condition that comprises different groups of heterogeneous diseases and has an increasing global prevalence^1,2^. To provide high quality care for people with DM, a patient-centred approach with a particular attention to patient and public involvement is a fundamental principle^3^. In early stages of diabetes research processes, patient-centredness can be achieved by incorporating the concerns, needs, and values of the stakeholders in the research agenda^4–6^.

The research priorities of physicians and scientists do not always match the needs and preferences of patients^5,7,8^, which remain insufficiently addressed^9^. A cluster-randomized controlled trial showed that when patients are involved, priorities for healthcare improvement can change and a higher level of agreement between patients and physicians can be achieved^10^. According to the Patient-Centered Outcomes Research Institute, the term ‘research priority setting’ includes the following engagement activities: ‘provide input on the research topic, prioritization/agenda setting and how to frame the research question’^11^. Patients are seen as experts on the disease through their individual experiences in the healthcare system^12^.

Previous studies have shown that people with DM have a high interest in diabetes research^13,14^. A recent review by Harris et al.^15^ investigated health outcomes in patient and community involvement in diabetes research projects. The main benefits of patient and public involvement on the research agenda were ‘initiating the research topic’ and ‘identifying different research questions’. Furthermore, several studies involved different groups of stakeholders (e.g. people with type 1 diabetes (T1DM), type 2 diabetes (T2DM) or other types of diabetes; relatives and health care providers) and used different study designs (e.g. mixed-methods and participatory approaches) to identify priorities in diabetes research^3,4,8,9,16–27^. However, it is still unclear how diverse these priorities are within the DM community. Brown et al.^17^ suggest that the individual perspectives of patients (e.g. determined by age^26^) have an influence on research priorities. Depending on the method used, there would be a risk for under-representing particular groups when setting research priorities (e.g. people with multiple comorbidities)^8,16,19–21,25^. In Germany, there was only one questionnaire study in 2011^23^ that analyzed the research priorities of people with DM. However, it used a convenience sample from the readership of a popular German news magazine and did not investigate the differences between groups of people with different sociodemographic or diabetes-related characteristics.

## Methods

The present questionnaire study aims to investigate (i) the importance and priorities of future diabetes-related research objectives for people with T1DM and T2DM in Germany; (ii) different subgroups of people with specific diabetes-related research priorities; and (iii) associated characteristics of the identified subgroups, such as sociodemographic and diabetes-related characteristics.

### Study design and population

This quantitative cross-sectional study was conducted from September 2018 to November 2018. Data were collected using a postal questionnaire among a nationwide random patient sample of a German statutory health insurance (‘*pronova BKK*’). The inclusion criteria for a diabetes diagnosis based on health insurance data were adapted from other studies^28,29^. People between the ages of 18 and 80 years were included if they had ‘(i) a regular documentation of 10th International Classification of Diseases (ICD-10) diagnosis “diabetes” (E10–E14) in three of four quarters in 2016 or (ii) regular prescription of antihyperglycaemic drugs (Anatomical-Therapeutic-Chemical classification A10) at least two prescriptions in 2016 or (iii) a single prescription of an antihyperglycaemic drug 2016 and a diagnosis of “diabetes” or a single prescription of an antihyperglycaemic drug within 2016 and a blood glucose or hemoglobin A_1c_ (HbA_1c_) measurement in the same quarter’^28^. Patients who had a caregiver, a care level 2–5, ICD F70–79 code (mental retardation) or ICD Z51.5 code (palliative care) were excluded.

The five-page questionnaire was sent out once via the health insurance by post to 3000 people (T1DM: n = 1000, mean age 53.6 years, 33.9% female; T2DM: n = 2000, mean age 65.3 years, 33.9% female, diabetes medication 69.9%). The questionnaires were returned to the Institute of Health Services Research and Health Economics by means of an enclosed prepaid envelope. Finally, a total of 869 people responded (29% response).

### Assessment of the importance and priorities of diabetes-related research objectives

The questionnaire intended to measure the importance of and priorities for future diabetes research objectives^30^ was based on a literature review and five focus groups consisting of people with T1DM and T2DM^30^. The focus group data were analyzed by a multidisciplinary, professional team (including several physicians, a psychologist, a nurse and several health economists) using qualitative inductive content analysis. As a result, the authors developed a questionnaire that included 18 diabetes-related research objectives. In a pretest phase, experts in the field of DM and questionnaire design were consulted, and cognitive pretests were conducted with patients treated by specialists in diabetes and with people from a diabetes self-help group. In addition, a final standard field pretest was conducted (n = 27). The final questionnaire can be found in Supplementary Material.

In the first section of the questionnaire, people with DM were asked the following: ‘How important is it for you personally that diabetes research accomplishes the following results in the next few years?’. Each of the 18 objectives could be answered on a 4-point Likert scale from ‘not very important’ to ‘extremely important’. In the second section of the questionnaire, the respondents were to indicate up to three of the 18 research objectives as their priorities. In our study, these 18 objectives were grouped into the following seven overarching topics (Supplementary Table S1): ‘treatment with the aim to cure diabetes’, ‘simplifying diabetes handling’, ‘stress reduction’, ‘prevention of long-term complications’, ‘prevention of acute complications’, ‘diabetes prevention’, and ‘information and personal responsibility’. A priority for the topic was assumed if at least one of the corresponding research objectives was chosen in the second section of the questionnaire.

### Assessment of associated characteristics

We collected age (in years), sex and education as sociodemographic factors. Education was assessed by reference to the highest school graduation, which was dichotomized and coded as ‘other graduation’ (including those without a school graduation) or ‘university entrance qualification’. In addition, we included the type of diabetes coded as ‘type 1 diabetes’ and ‘type 2 diabetes’. The code ‘unknown’ was excluded from statistical analysis. Diabetes duration was measured by the question ‘How long have you had diabetes?’, and HbA_1c_ values were evaluated in percent by asking ‘What was your last HbA_1c_?’. Alternatively, the respondent had the option to mark ‘I don’t know’. HbA_1c_ values were categorized based on the guidelines for diabetes treatment and coded as ‘ < 6.5%’, ‘6.5% to < 7.5%’, and ‘ ≥ 7.5%’^31,32^. Concerning diabetes treatment, participants were asked whether they injected insulin (yes/no) and whether they were primarily treated by a general practitioner or a diabetologist.

### Statistical analysis

We obtained descriptive summaries for participant characteristics depending on the distribution of the variables by frequencies, percentages, means (M), standard deviations (SD), median and interquartile range. Data were analyzed and presented separately for the two types of diabetes, T1DM and T2DM. Latent class analyses (LCAs) were performed with Mplus Version 8.4 (Muthén & Muthén, 1998–2020); the remaining quantitative analyses were performed with SAS 9.4.

#### Analysis of diabetes-related research priorities using latent class analysis (LCA)

LCA is a statistical approach used to identify a finite set of mutually exclusive and exhaustive unobserved (latent) classes of individuals based on their pattern of observed multivariate categorical variables. To identify the optimal number of subgroups with specific diabetes-related research priorities and associated characteristics, LCAs without covariates were performed. We modelled latent classes with one to six classes based on the merged seven topics. In line with Lanza et al.^33^, we chose the best model by analyzing whether the classes were meaningful and had a minimum class prevalence of 5%. In addition, we also took into account the ‘law of parsimony’^34^ and considered the following indicators for model fit: Lower values of the Bayesian information criterion (BIC) indicated better fit. A (relative) entropy close to ‘one’ indicated high separation of classes.

Finally, we performed LCA models for T1DM and T2DM with the favored number of classes including the following variables: age, sex, education, diabetes duration, HbA_1c_ values (including the answer ‘I don't know’), insulin injection and primary practitioner. The variables ‘insulin injection’ and ‘primary practitioner’ were excluded in models involving participants with T1DM, as T1DM patients are generally treated with insulin and in specialized practices by diabetologists.

### Ethics approval and consent to participate

The present study was approved by the ethics committee of the Heinrich Heine University Düsseldorf (study reference number 5651, date of decision: April 06, 2017). The study was performed in accordance with the Declaration of Helsinki. Informed consent was obtained from all individual participants included in the study.

## Results

### Participant characteristics

Table 1 shows the characteristics of the 869 participants, 295 of whom were diagnosed with T1DM and 523 with T2DM. Of those with T1DM, one-third were female and almost half had a university entrance qualification. The average duration of diabetes was about 21 years. Nearly all participants with T1DM reported being treated with insulin, predominantly by a diabetologist. Among those with T2DM, one-third was female, about one in five had a university entrance qualification, and the average duration of diabetes was about 13 years. Approximately 37% of participants with T2DM were taking insulin and about one-quarter were treated primarily by a diabetologist. Compared to the entire sample (T1DM, n = 1000; T2DM, n = 2000), the mean age of the respondents was slightly lower for those with T1DM (47.9 years vs. 53.6 years) and slightly higher for those with T2DM (68.4 years vs. 65.3 years). The percentage of female participants was similar in both groups (T1DM: 33.2% vs. 33.9%; T2DM: 29.6% vs. 33.9%).

**Table 1:** Participants’ characteristics.

Characteristics		N (%)/M ± SD; median (quartile)	
		Type 1 diabetes	Type 2 diabetes
Number of participants		295	523
Age (years), n = 294/514		47.9 ± 16.9; 49.0 (q1: 33.0; q3: 62.0)	68.4 ± 8.6; 70.0 (q1: 63.0; q3: 76.0)
Sex, n = 295/523	Female	98 (33.2)	155 (29.6)
Education, n = 293/512	No graduation	2 (0.7)	38 (7.4)
	Other graduation	154 (52.6)	378 (73.8)
	University entrance qualification	137 (46.7)	96 (18.8)
Diabetes duration, n = 292/504		21.4 ± 13.7; 18.5 (q1: 10.0; q3: 31.0)	12.6 ± 8.8; 10.0 (q1: 6.0; q3: 17.0)
Insulin, n = 294/522		289 (98.3)	191 (36.6)
Treated primarily by a diabetologist, n = 286/500		268 (93.7)	129 (25.8)
HbA_1c_ (%), n = 280/420		7.1 ± 0.9; 7.0 (q1: 6.5; q3: 7.5)	6.9 ± 0.9; 6.8 (q1: 6.3; q3: 7.4)
n = 293/501	< 6.5%	61 (20.8)	127 (25.3)
	6.5 to < 7.5%	142 (48.5)	200 (39.9)
	≥ 7.5%	77 (26.3)	93 (18.6)
	Unknown	13 (4.4)	81 (16.2)

*M* = mean, *SD =* standard deviation, *HbA*_*1c*_ = hemoglobin A1c.

### Importance and priorities of objectives concerning future diabetes research

**Figure 1:**
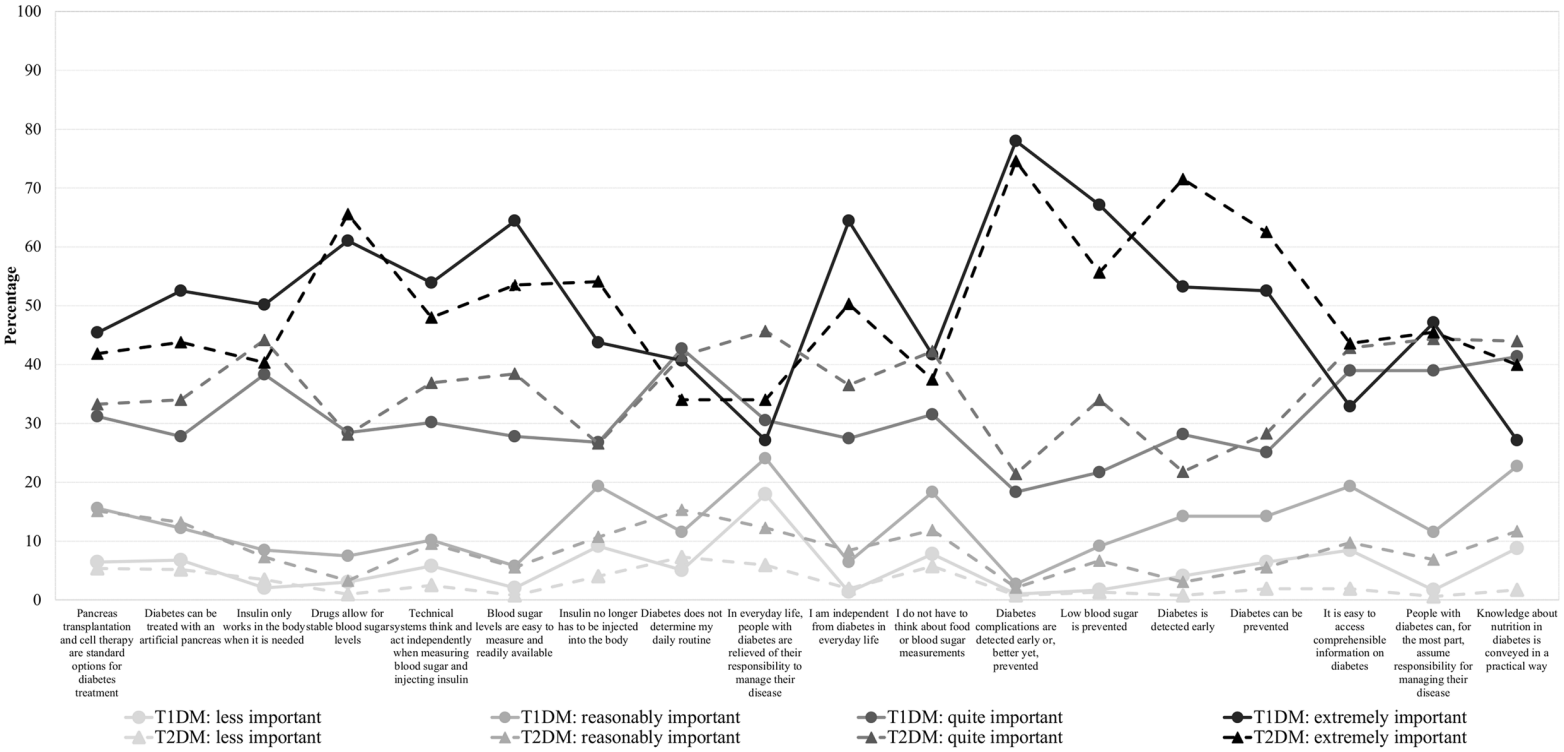
Importance of objectives concerning future diabetes research stratified by type of diabetes (T1DM n = 295, T2DM n = 523).

Respondents with T1DM have most often rated the objective ‘diabetes complications are detected early or, better yet, prevented’ as very or extremely important for future diabetes research (96.3%) (Fig. 1). In addition, more than 90% of respondents with T1DM indicated the research objectives ‘blood sugar levels are easy to measure and readily available’ and ‘I am independent from diabetes in everyday life’ as very or extremely important. In contrast, the research objective ‘in everyday life, people with diabetes are relieved of their responsibility to manage their disease’ was less frequently rated as very or extremely important (57.6%).

**Figure 2:**
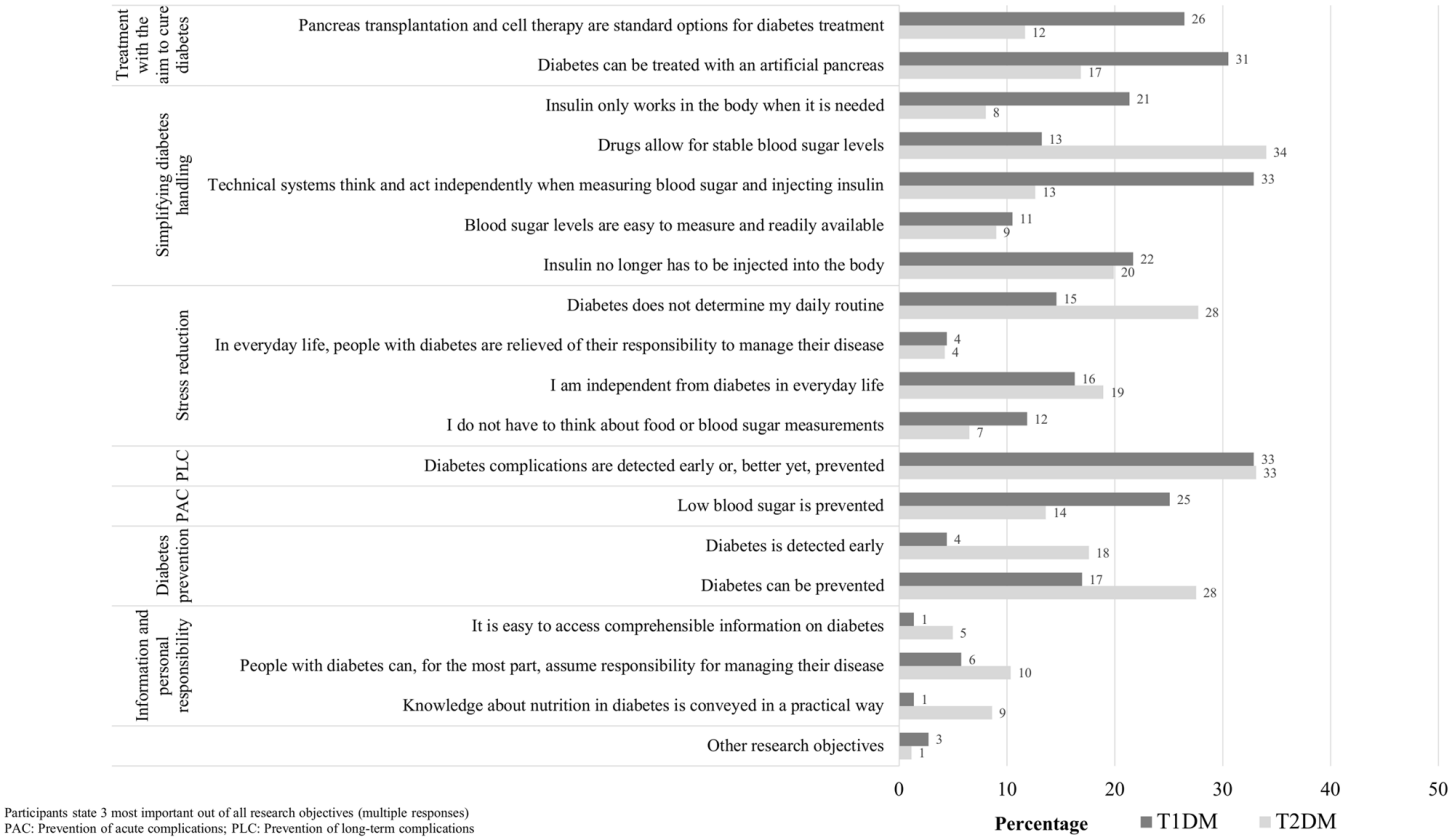
Priorities of people with T1DM (n = 295) and T2DM (n = 523) regarding future diabetes-related research objectives.

When asked to identify the top three research objectives, the majority of respondents with T1DM selected ‘diabetes complications are detected early or, better yet, prevented’ (32.9%) and ‘technical systems think and act independently when measuring blood sugar and injecting insulin’ (32.9%) (Fig. 2). The research objectives ‘it is easy to access comprehensible information on diabetes’ and ‘knowledge about nutrition in diabetes is conveyed in a practical way’ were rarely selected (1.4%, respectively).

Respondents with T2DM have also most often rated the research objective ‘diabetes complications are detected early or, better yet, prevented’ as very or extremely important (96.0%) (Fig. 1). Furthermore, over 90% of respondents with T2DM indicated the following research objective to be very or extremely important: ‘drugs allow for stable blood sugar levels’, ‘diabetes is detected early’, ‘blood sugar levels are easy to measure and readily available’ and ‘diabetes can be prevented’. The research objective ‘pancreas transplantation and cell therapy are standard options for diabetes treatment’ was less frequently rated as very or extremely important (75.1%).

When asked to identify the top three research objectives, the majority of respondents with T2DM selected the following two: ‘drugs allow for stable blood sugar levels’ (34.0%) and ‘diabetes complications are detected early or, better yet, prevented’ (33.1%) (Fig. 2). The research objectives ‘in everyday life, people with diabetes are relieved of their responsibility to manage their disease’ (4.2%) and ‘it is easy to access comprehensible information on diabetes’ were rarely selected (5.0%).

### Priorities on research topics that include future diabetes-related research objectives

**Figure 3:**
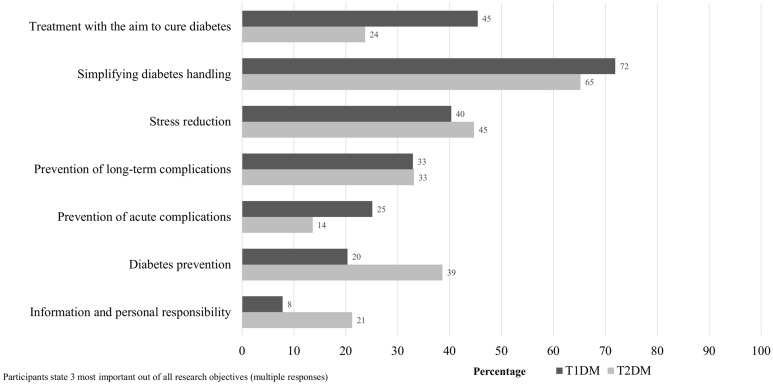
Priorities of people with T1DM (n = 295) and T2DM (n = 523) regarding categories of future diabetes-related research objectives.

When grouping the research objectives into seven research topics, we see that most respondents with T1DM listed at least one objective on ‘simplifying diabetes handling’ (71.9%) among the top three (Fig. 3). Research objectives concerning ‘diabetes prevention’ (20.3%) and ‘information and personal responsibility’ (7.8%) were mentioned less frequently.

Most respondents with T2DM also selected at least one research objective on ‘simplifying diabetes handling’ (65.2%). Research objectives concerning ‘information and personal responsibility’ (21.2%) and ‘prevention of acute complications’ (13.6%) were mentioned less frequently.

### Subgroups with specific priorities regarding future diabetes-related research objectives

The analysis showed that a three-class model exhibited the best fit for people with T1DM and T2DM. This model had the lowest BIC value and the best entropy score. The model fit indicators are described in detail in Supplementary Table S2.

**Figure 4:**
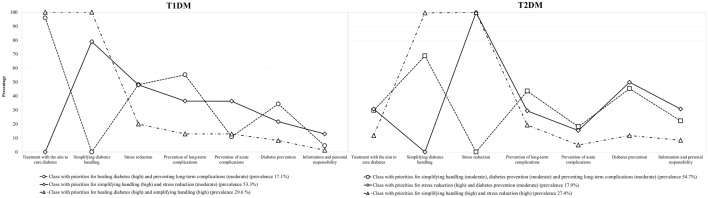
LCA with 3 classes and covariates (T1DM, n = 287, T2DM, n = 435).

For people with T1DM, we identified the following three research priority profiles (Fig. 4):

- *High priority for the research topic ‘treatment with the aim to cure diabetes’ and moderate priority for the research topic ‘prevention of long-term complications (class with priorities for healing diabetes (high) and preventing long-term complications’ (moderate)):* This class included participants most likely to prioritize research objectives concerning ‘treatment with the aim to cure diabetes’ (96.0%). Here, the topic ‘prevention of long-term complications’ had the probability of 55.3%. For the other topics, the probability was between 0.0% and 48.2%. Overall, the estimated class prevalence was 17.1%.
- *High priority for the research topic ‘simplifying diabetes handling’ and moderate priority for the research topic ‘stress reduction’ (class with priorities for simplifying handling (high) and stress reduction (moderate)):* This class included participants most likely to prioritize research objectives concerning ‘simplifying diabetes handling’ (79.1%). For ‘stress reduction’, the probability was 48.0%. For other topics, it lay between 0.0% and 36.5%. The estimated class prevalence was 53.3%.
- *High priority for the research topic ‘treatment with the aim to cure diabetes’ and for ‘simplifying diabetes handling’* (*class with priorities for healing diabetes (high) and simplifying handling (high)*): This class included participants who prioritize research objectives on the topics ‘treatment with the aim to cure diabetes’ and ‘simplifying diabetes handling’ (100.0% respectively). For other topics, the probability was between 1.2% and 20.0%. The estimated class prevalence was 29.6%.

For people with T2DM, we identified the following three research priority profiles (Fig. 4):

- *Moderate priority for the research topics ‘simplifying diabetes handling’, ‘diabetes prevention’ and ‘prevention of long-term complications’ (class with priorities for simplifying handling (moderate), diabetes prevention (moderate), and preventing long-term complications (moderate)):* This class included participants most likely to prioritize research objectives on the topic ‘simplifying diabetes handling’ (68.9%). For the topics ‘diabetes prevention’ and ‘prevention of long-term complications’, the probability was 45.4% and 43.7%. For the others, the probability was between 0.0% and 29.8%. The estimated class prevalence was 54.7%.
- *High priority for the research topic ‘stress reduction’ and moderate priority for the research topic ‘diabetes prevention’ (class with priorities for stress reduction (high) and diabetes prevention (moderate)):* This class included participants who prioritize research objectives on the topic ‘stress reduction’ (100%). In addition, half prioritize research objectives concerned with ‘diabetes prevention’ (50%). For the others, the probability was between 0.0% and 30.8%. The estimated class prevalence was 17.9%.
- *High priority for the research topics ‘simplifying diabetes handling’ and ‘stress reduction’ (class with priorities for simplifying handling (high) and stress reduction (high)):* This class included participants who prioritize research objectives on the topics ‘simplifying diabetes handling’ and ‘daily stress’ (100% respectively). For other topics, the probability was between 5.0% and 19.3%. The estimated class prevalence was 27.4%.

### Associated characteristics of the identified subgroups

We found significant associations between belonging to one of the identified subgroups and age as well HbA_1c_-values. In T1DM, the class ‘*priorities for healing diabetes (high) and preventing long-term complications (moderate)’* was younger than the class ‘*priorities for simplifying handling (high) and stress reduction (moderate)’* (Table 2). In addition, this class was more likely to have intermediate (6.5% to < 7.5%) than lower (< 6.5%) HbA_1c_ values compared with the class *‘priorities for simplifying handling (high) and stress reduction (moderate)’* and was more likely to have intermediate or higher (≥ 7.5%) than lower HbA_1c_ values compared with the class ‘*priorities for healing diabetes (high) and simplifying handling (high)*’. The class *‘priorities for simplifying handling (high) and stress reduction (moderate)’* was older than the other two and more likely to have lower than intermediate/unknown HbA_1c_ values compared with the classes ‘*priorities for healing diabetes (high) and preventing long-term complications (moderate)’/ ‘priorities for healing diabetes (high) and simplifying handling (high)’.*

**Table 2:** LCA with covariates stratified by type of diabetes.

	T1DM (n = 287)		
	*‘class with priorities for simplifying handling (high) and stress reduction (moderate)’ vs. ‘class with priorities for healing diabetes (high) and preventing long-term complications (moderate)’’*	***‘*** *class with priorities for healing diabetes (high) and simplifying handling (high)’ vs. ‘class with priorities for healing diabetes (high) and preventing long-term complications (moderate)’’’*	*‘class with priorities for healing diabetes (high) and simplifying handling (high)’ vs. ‘class with priorities for simplifying handling (high) and stress reduction (moderate)’’’*
	OR [95% CI]	OR [95% CI]	OR [95% CI]
Age (years)	**1.05 [1.01; 1.08]**	1.03 [0.99; 1.06]	**0.98 [0.96; 1.00]**
Sex (female)	1.10 [0.50; 2.40]	1.86 [0.82; 4.20]	1.70 [0.94; 3.06]
Education (university entrance qualification)	1.44 [0.62; 3.33]	1.99 [0.85; 4.65]	1.38 [0.75; 2.54]
Diabetes duration (years)	0.99 [0.97; 1.02]	1.01 [0.98; 1.04]	1.02 [0.99; 1.04]
HbA_1c_	**0.18 [0.05; 0.60]**	**0.16 [0.05; 0.58]**	0.91 [0.45; 1.84]
*(6.5 to < 7.5% vs. < 6.5%)*			
*(≥ 7.5% vs. < 6.5%)*	0.38 [0.09; 1.54]	**0.21 [0.05; 0.90]**	0.56 [0.25; 1.22]
*(unknown vs. < 6.5%)*	0.17 [0.02; 1.38]	0.89 [0.10; 7.68]	**5.29 [1.12; 24.13]**

	T2DM (n = 435)		
	*‘class with priorities for stress reduction (high) and diabetes prevention (moderate)’ vs. ‘class with priorities for simplifying handling (moderate), diabetes prevention (moderate) and preventing long-term complications (moderate)’*	*‘class with priorities for simplifying handling (high) and stress reduction (high)’ vs. ‘class with priorities for simplifying handling (moderate), diabetes prevention (moderate) and preventing long-term complications (moderate)’*	*‘class with priorities for simplifying handling (high) and stress reduction (high)’ vs. ‘class with priorities for stress reduction (high) and diabetes prevention (moderate)’*
	OR [95% CI]	OR [95% CI]	OR [95% CI]
Age (years)	0.98 [0.95; 1.01]	**0.96 [0.94; 0.99]**	0.98 [0.95; 1.02]
Sex (female)	0.97 [0.55; 1.71]	1.18 [0.73; 1.91]	1.22 [0.64; 2.29]
Education (university entrance qualification)	1.44 [0.75; 2.75]	1.36 [0.77; 2.40]	0.95 [0.48; 1.87]
Insulin (yes)	0.82 [0.45; 1.51]	1.09 [0.65; 1.83]	1.33 [0.69; 2.56]
Treated primarily by a diabetologist	0.67 [0.33; 1.34]	0.63 [0.38; 1.07]	0.95 [0.46; 1.98]
Diabetes duration (years)	0.99 [0.96; 1.03]	1.01 [0.98; 1.04]	1.02 [0.98; 1.06]
HbA_1c_	0.88 [0.45; 1.70]	0.89 [0.50; 1.60]	1.01 [0.48; 2.12]
*(6.5 to < 7.5% vs. < 6.5%)*			
*(≥ 7.5% vs. < 6.5%)*	0.81 [0.35; 1.89]	1.40 [0.72; 2.72]	1.72 [0.70; 4.20]
*(unknown vs. < 6.5%)*	1.57 [0.74; 3.35]	1.18 [0.56; 2.49]	0.75 [0.31; 1.82]

OR = odds ratio (corresponding to one unit change in age and diabetes duration).

CI = confidence interval.

At level 5% significant results are in bold.

In T2DM, classes differed significantly in age. The class ‘*priorities for simplifying handling (high) and stress reduction (high)’* was younger than the class ‘*priorities for simplifying handling (moderate), diabetes prevention (moderate) and preventing long-term complications (moderate)*’. Respondents’ characteristics stratified by classes are presented in Table 3 and Supplementary Table S3.

**Table 3:** Description of class characteristics compared to the other classes.

Selected characteristics	Typical person with T1DM and…		
	priorities for healing diabetes (high) and preventing long-term complications (moderate)	priorities for simplifying handling (high) and stress reduction (moderate)	priorities for healing diabetes (high) and simplifying handling (high)
Age	*Younger*	*Older*	*Younger*
Sex	Fewer women	Fewer women	More women
Education	Lower education	Lower education	Higher education
Diabetes duration	Shorter duration	Longer duration	Longer duration
HbA_1c_	*Rather poor*	*Rather good*	*Rather good/unknown*

Characteristics	Typical person with T2DM and…		
	priorities for simplifying handling (moderate), diabetes prevention (moderate) and preventing long-term complications (moderate)	priorities for stress reduction (high) and diabetes prevention (moderate)	priorities for simplifying handling (high) and stress reduction (high)
Age	*Older*	Younger	*Younger*
Sex	Fewer women	Fewer women	More women
Education	Lower education	Higher education	Higher education
Insulin	Usually, yes	Usually, no	Usually, yes
Treated primarily by	Usually, diabetologist	Usually, physician	Usually, diabetologist
Diabetes duration	Longer duration	Shorter duration	Longer duration
HbA_1c_	Rather good	Rather good/unknown	Rather poor

The results were extracted from the LCA analysis (Table 2 and Supplementary Table S3).

At level 5% significant results (Table 2) are marked with italics.

## Discussion

### Main findings

Most respondents rated all future research objectives as at least very important, especially ‘diabetes complications are detected early or, better yet, prevented’. Research priorities primarily concerned research objectives related to the topic ‘simplifying diabetes handling’. Comparing T1DM and T2DM, people with T1DM assigned higher priority to the research topic ‘treatment with the aim of curing diabetes’ and people with T2DM to the research topic ‘diabetes prevention’. The following three classes of people with T1DM with different research priority profiles were identified: ‘*class with priorities for healing diabetes (high) and prevention of long-term complications (moderate)’ (prevalence 17.1%), ‘class with priorities for simplifying handling (high) and stress reduction (moderate)’ (53.3%), ‘class with priorities for healing diabetes (high) and simplifying handling (high)’ (29.6%)*. They differed significantly in age and HbA_1c_ values. People with T2DM could be assigned to the following three classes: *‘class with priorities for simplifying handling (moderate), diabetes prevention (moderate), and prevention of long-term complications (moderate)’* (54.7%), *‘class with priorities for stress reduction (high) and diabetes prevention (moderate)’* (17.9%), and *‘class with priorities for simplifying handling (high) and stress reduction (high)’* (27.4%)*.* Two of them differed significantly in age.

### Discussion of the findings and comparison to other studies

A high importance of the research objective ‘diabetes complications are detected early or, better yet, prevented’ was also found in other studies on T1DM and T2DM^3,18,19,25^. The present results showed that while this objective pertaining to the research topic ‘prevention of long-term complications’ matter greatly, other research topics were given even higher priority when objectives were grouped. In particular, respondents reported research priorities on ‘simplifying diabetes handling’, e.g. simple ways to measure blood glucose, which were also clearly expressed in previous work^23,30^. As discussed by other authors^25,30^, one explanation could be that research priorities of people with DM reflect basic needs and are therefore predominantly focused on short-term solutions that directly affect their current daily lives. A dialogue about different research priorities can increase the relevance of diabetes research, e.g. by raising awareness of everyday issues^15^. The findings support the recommendations of other studies^4–6^ that research institutes and funders should involve patients more in decision-making about the research direction.

Differences in research priorities between respondents with T1DM and T2DM may be due to the heterogeneous health conditions, e.g., the underlying cause of increase in blood glucose levels^2^. In T1DM, prioritization of research objectives on the research topic ‘treatment with the aim to cure diabetes’ was also mentioned by others^18^ and may occur due to frequent life-threatening complications^2^ and lifelong insulin treatment^35^. In future, T1DM could be cured through biological approaches such as pancreas transplantation^36,37^. Respondents’ research priorities may be influenced by new scientific advances expected in the search for a cure in the coming years^36^. In T2DM, prioritization of research objectives on the research topic ‘diabetes prevention’ may be influenced by knowledge of several effective (lifestyle) interventions that have been shown to prevent T2DM^38–40^. Therefore, prevention is also of great importance in medical care^39^. Our findings were new compared to the previous German study where differences between diabetes types were not investigated^23^. Comparing the research priorities of the two studies, we see that the priorities identified by Arnolds et al.^23^ in relation to the research topics ‘treatment with the aim to cure diabetes’ and ‘simplifying diabetes handling’ are more in line with the present profiles for T1DM. Arnolds et al.^23^ found that research on psychological aspects and trainings were prioritized less frequently, while these had a higher priority in our study, especially for people with T2DM (e.g., within the research topic ‘information and personal responsibility’). In the light of our findings, we recommend considering the research priorities of people with T1DM and T2DM separately.

Although different research priority profiles were identified in people with T1DM and T2DM, the classes did not differ significantly in the characteristics collected (except for age and HbA_1c_ values). Therefore, this study was not able to identify ‘types’ of people^41^ with specific research priorities and characteristics. The question arises whether the differences can be explained by other factors not collected in the present study, e.g., cultural background^17^ and comorbidities^8,16,19,21,25^. Nevertheless, being aware of the different research priorities is important when involving people with T1DM and T2DM more specifically in research projects.

In T1DM, the largest and oldest class indicated research priorities for simplifying handling (high) and stress reduction (moderate) rather than for healing DM, as in the other two classes. This is surprising given that Gadsby^18^ defined finding a cure as the overarching, long-term research priority in T1DM. One reason may lie in personal attitudes, as middle-aged persons with T1DM reported that they were less focused on hoping for a future cure and more on taking responsibility for their own diabetes management^42^. In contrast, the research topic ‘stress reduction’ was crucial addressed by the largest class in T1DM described here, which was also listed by Gadsby et al.^18^. Boddy et al.^8^ emphasize the importance of such research topics for those affected, noting that these have often been neglected in funded research agendas^8^. The present results confirm the importance of involving a diverse group of people with different research priorities in setting research agendas to ensure that their varied interests are considered.

In T2DM, two of three classes that included most participants had priorities for simplifying DM handling, although insulin use was less common in T2DM than in T1DM. Nevertheless, those research objectives were of key priority, particularly the wish that ‘drugs allow for stable blood sugar levels’. As has already been shown^43^, achieving adequate HbA_1c_ levels is challenging for people with T2DM. Although no significant differences in HbA_1c_ values were found among priority classes, problems during the course of treatment leading to a change in treatment strategy may have affected the priorities reported. Further longitudinal studies should investigate possible changes in priorities over time, such as when DM treatment is adjusted (e.g., from no antihyperglycemic medication to medication with oral glucose-lowering drugs).

### Limitations and strengths

The major limitation is the low response rate (29.0%), which could have led to a non-responder bias. We are aware of studies with higher, but also with lower response rate (51.1%^44^; 8.1%^45^) among members of statutory health insurance. However, we performed a responder/non-responder analysis. The age and sex distribution were similar for the responders and the entire sample. But there are more people with T2DM who are treated with insulin in our sample compared to a representative study in 2010 (36.6% vs. 25.2%)^46^. Therefore, people who have received treatment specifically targeting lifestyle changes may be underrepresented. This could be due to the fact that people with DM who are treated with insulin and are therefore more likely to be affected by DM in their daily lives are more interested in participating in the study. Another limitation is the language. The questionnaire was provided only in German. A few recipients responded apologetically that they could not fill out the questionnaire due to insufficient comprehension. In future studies, the questionnaire could be translated into multiple languages to reach more participants from diverse cultural backgrounds. Another limitation is that we possibly did not capture the relevant associated factors (e.g., cultural background, comorbidities) that explain the differences among the identified research priority profiles for T1DM and especially for T2DM.

A strength of the study is the questionnaire used, which was developed using a qualitative and participatory approach based on the views of people with DM. In addition, the survey enabled us to reach a large number of people with T1DM and T2DM with different sociodemographic and clinical backgrounds nationwide. Other studies use various participatory and collaborative methods to set priorities, for example the SEED method^4^, the James Lind Alliance approach^3,18^, and multi-voting^20^. Clavisi et al.^5^ reported that using only one method can limit the findings of prioritized questions. In addition, they recommended to involve further relevant stakeholders (e.g. clinicians, policy makers) when dealing with the challenge covering clinical and non-clinical research questions as broadly as possible^5^. We see our work as a first step towards a joint dialogue between patients and researchers as reported by Abma and Broerse^47^. Further discussions can build on our results.

## Conclusions

Knowledge of diabetes-related research priorities enables researchers to improve their research on behalf of people with DM and to align research with their needs and wishes. In particular, objectives related to simplifying DM handling are important for patients. This study has shown that groups of people with T1DM and T2DM have divergent research priorities, focusing to different degrees on healing diabetes, prevention of long-term complications, diabetes prevention, stress reduction and simplifying handling. This highlights the importance of including both people with T1DM and people with T2DM more specifically in research projects. In addition, we join the current calls for a diverse group of people (with different research priority profiles) to be actively involved in setting research agendas to ensure that their varied interests are considered in research programs overall. Further research should investigate possible changes of research priorities during the course of the disease (e.g., through changes in diabetes treatment) and include the research priorities of other relevant stakeholders (e.g., physicians) to promote a broad collection of research priorities and a joint, cross-perspective dialogue. Research pathways can then be determined based on a reconciliation of what people with DM desire and what researchers deem important. In this way, both needs-based research on potentially short-term solutions and basic research or research on medium- and long-term solutions that may be prioritized by a group of scientists can be included equally in a common prioritization process.

## Supplementary Information

Supplementary Information.

## Data Availability

Data available on request from the corresponding author due to privacy/ethical restrictions.
