# Supplementary material for: Diabetes-related research priorities of people with type 1 and type 2 diabetes: a cross-sectional study in Germany
Source: Sci Rep. 2022 Dec 2;12:20835. doi: 10.1038/s41598-022-24180-y (PMC9718826; doi:10.1038/s41598-022-24180-y)
Supplement: Supplementary file 1 — Supplementary Information. [file 41598_2022_24180_MOESM1_ESM.docx]

**Supplementary Material: Diabetes Research Questionnaire**

How **important** is it for you personally that diabetes research accomplishes the following results in the next few years?

*Please tick only one box per line.*

| *How important is it for research to achieve the following for you in the future?* | *not very important* | *somewhat important* | *very important* | *extremely important* |
| --- | --- | --- | --- | --- |
| Diabetes does not determine my daily routine. | ❑ | ❑ | ❑ | ❑ |
| Insulin only works in the body when it is needed. | ❑ | ❑ | ❑ | ❑ |
| Diabetes can be prevented. | ❑ | ❑ | ❑ | ❑ |
| Pancreas transplantation and cell therapy are standard options for diabetes treatment. | ❑ | ❑ | ❑ | ❑ |

*Please tick only one box per line.*

| *How important is it for research to achieve the following for you in the future?* | *not very important* | *somewhat important* | *very important* | *extremely important* |
| --- | --- | --- | --- | --- |
| In everyday life, people with diabetes are relieved of their responsibility to manage their disease. | ❑ | ❑ | ❑ | ❑ |
| It is easy to access comprehensible information on diabetes. | ❑ | ❑ | ❑ | ❑ |
| Diabetes can be treated with an artificial pancreas. | ❑ | ❑ | ❑ | ❑ |
| Low blood sugar is prevented. | ❑ | ❑ | ❑ | ❑ |

Please rate the additional research goals that are listed on the next page:

**Please turn over!** 1 of 5

*Please tick only one box per line.*

| *How important is it for research to achieve the following for you in the future?* | *not very important* | *somewhat important* | *very important* | *extremely important* |
| --- | --- | --- | --- | --- |
| Drugs allow for stable blood sugar levels. | ❑ | ❑ | ❑ | ❑ |
| Technical systems think and act independently when measuring blood sugar and injecting insulin. | ❑ | ❑ | ❑ | ❑ |
| I am independent from diabetes in everyday life. | ❑ | ❑ | ❑ | ❑ |
| Knowledge about nutrition in diabetes is conveyed in a practical way. | ❑ | ❑ | ❑ | ❑ |
| Diabetes is detected early. | ❑ | ❑ | ❑ | ❑ |

*Please tick only one box per line.*

| *How important is it for research to achieve the following for you in the future?* | *not very important* | *somewhat important* | *very important* | *extremely important* |
| --- | --- | --- | --- | --- |
| Diabetes complications are detected early or, better yet, prevented. | ❑ | ❑ | ❑ | ❑ |
| Blood sugar levels are easy to measure and readily available. | ❑ | ❑ | ❑ | ❑ |
| I do not have to think about food or blood sugar measurements. | ❑ | ❑ | ❑ | ❑ |
| People with diabetes can, for the most part, assume responsibility for managing their disease. | ❑ | ❑ | ❑ | ❑ |
| Insulin no longer has to be injected into the body. | ❑ | ❑ | ❑ | ❑ |

Please continue on the next page:

**Please turn over!** 2 of 5

You will be shown the same research goals as above.

***Please tick only the 3 research goals that are most important to you!***

| A | 🔾 | Diabetes does not determine my daily routine. |
| --- | --- | --- |
| B | 🔾 | Insulin only works in the body when it is needed. |
| C | 🔾 | Diabetes can be prevented. |
| D | 🔾 | Pancreas transplantation and cell therapy are standard options for diabetes treatment. |
| E | 🔾 | In everyday life, people with diabetes are relieved of their responsibility to manage their disease. |
| F | 🔾 | It is easy to access comprehensible information on diabetes. |
| G | 🔾 | Diabetes can be treated with an artificial pancreas. |
| H  I | 🔾 | Low blood sugar is prevented. |
| I | 🔾 | Drugs allow for stable blood sugar levels. |
| J | 🔾 | Technical systems think and act independently when measuring blood sugar and injecting insulin. |
| K | 🔾 | I am independent from diabetes in everyday life. |
| L | 🔾 | Knowledge about nutrition in diabetes is conveyed in a practical way. |
| M | 🔾 | Diabetes is detected early. |
| N | 🔾 | Diabetes complications are detected early or, better yet, prevented. |
| O | 🔾 | Blood sugar levels are easy to measure and readily available. |
| P | 🔾 | I do not have to think about food or blood sugar measurements. |
| Q | 🔾 | People with diabetes can, for the most part, assume responsibility for managing their disease. |
| R | 🔾 | Insulin no longer has to be injected into the body. |
| S | 🔾 | Other research goal:  _____________________________________________ *(please specify)* |

**Please turn over!** 3 of 5

| **Topic**  *(please enter the letter)* | | **I am particularly interested in these three topics:** |
| --- | --- | --- |
|  | |  |
|  |  |  |
|  |  |  |
|  |  |  |
|  |  |  |
|  |  |  |

1. **What is particularly important to you in diabetes research?**

***_________________________________________________________________________________________________________________***

***_________________________________________________________________________________________________________________***

***_________________________________________________________________________________________________________________***

***_________________________________________________________________________________________________________________***

***_________________________________________________________________________________________________________________***

Now just a few more details about yourself:

**Please turn over!** 4 of 5

**Thank you for completing this questionnaire!**

**Supplementary Table S1**

| *Items of future research objectives with corresponding topics from a total of seven* | | |
| --- | --- | --- |
| **Item** | **Future research objective** | **Topics** |
| **D** | Pancreas transplantation and cell therapy are standard options for diabetes treatment. | Treatment with the aim to cure diabetes |
| **G** | Diabetes can be treated with an artificial pancreas. | Treatment with the aim to cure diabetes |
| **B** | Insulin only works in the body when it is needed. | Simplifying diabetes handling |
| **I** | Drugs allow for stable blood sugar levels. | Simplifying diabetes handling |
| **J** | Technical systems think and act independently when measuring blood sugar and injecting insulin. | Simplifying diabetes handling |
| **O** | Blood sugar levels are easy to measure and readily available. | Simplifying diabetes handling |
| **R** | Insulin no longer has to be injected into the body. | Simplifying diabetes handling |
| **A** | Diabetes does not determine my daily routine. | Stress reduction |
| **E** | In everyday life, people with diabetes are relieved of their responsibility to manage their disease. | Stress reduction |
| **K** | I am independent from diabetes in everyday life. | Stress reduction |
| **P** | I do not have to think about food or blood sugar measurements. | Stress reduction |
| **N** | Diabetes complications are detected early or, better yet, prevented. | Prevention of long-term complications |
| **H** | Low blood sugar is prevented. | Prevention of acute complications |
| **M** | Diabetes is detected early. | Diabetes prevention |
| **C** | Diabetes can be prevented. | Diabetes prevention |
| **F** | It is easy to access comprehensible information on diabetes. | Information and personal responsibility |
| **Q** | People with diabetes can, for the most part, assume responsibility for managing their disease. | Information and personal responsibility |
| **L** | Knowledge about nutrition in diabetes is conveyed in a practical way. | Information and personal responsibility |
|  |  |  |

**Supplementary Table S2**

*Statistical parameters for models with different numbers of classes per LCA*

| **No. of classes** | **BIC** | **AIC** | **aBIC** | **ENTROPY** |
| --- | --- | --- | --- | --- |
| **1**  *T1DM*  *T2DM* | 2360.269  4329.659 | 2334,460  4299,842 | 2338,070  4307,439 | 1  1 |
| **2**  *T1DM*  *T2DM* | 2348.724  4307.506 | 2293,419  4243,612 | 2301,154  4259,893 | 1  1 |
| **3**  *T1DM*  *T2DM* | **2343.622**  **4282.564** | 2258,821  4184,594 | 2270,682  4209,557 | 1  1 |
| **4**  *T1DM*  *T2DM* | 2365.090  4301.274 | 2250,794  4169,227 | 2266,780  4202,873 | 1  1 |
| **5**  *T1DM*  *T2DM* | 2385.036  4318.528 | 2241,244  4152,404 | 2261,356  4194,732 | 1  0.973 |
| **6**  *T1DM*  *T2DM* | 2397.036  4342.832 | **2223,748**  **4142,631** | **2247,985**  **4193,643** | 0.975  0.971 |

Information criteria (smaller values indicate better fits, smallest values per LCA in bold). BIC = Bayesian information criterion, AIC= Akaike information criterion, aBIC= Sample size adjusted BIC, Entropy = relative entropy (values close to 0 indicate poor class separation (i.e. random guessing), values close to 1 indicating that classes are well-separated)

**Supplementary Table S3**

| *Participants’ characteristics* | | | | |
| --- | --- | --- | --- | --- |
| **Characteristics** | **T1DM** | priorities for healing diabetes (high) and preventing long-term complications (moderate) | priorities for simplifying handling (high) and stress reduction (moderate) | priorities for healing diabetes (high) and simplifying handling (high) |
|  | **T2DM** | priorities for simplifying handling (moderate), diabetes prevention (moderate) and preventing long-term complications (moderate) | priorities for stress reduction (high) and diabetes prevention (moderate) | priorities for simplifying handling (high) and stress reduction (high) |
|  |  | N (%) / M ± SD; median (quartile) | | |
| **Number of participants** | *T1DM*  *T2DM* | 47  238 | 155  78 | 85  119 |
|  |  |  |  |  |
| **Age** (years) | *T1DM*  *T2DM* | 41.0 ± 15.8  69.4 ± 8.3 | 50.8 ± 16.3  67.9 ± 8.2 | 45.9 ± 17.7  67.2 ± 9.0 |
|  |  |  |  |  |
| **Sex** (female) | *T1DM*  *T2DM* | 14 (29.8)  72 (30.3) | 48 (31.0)  22 (28.2) | 35 (41.2)  39 (32.8) |
|  |  |  |  |  |
| **Education** (university entrance qualification) | *T1DM*  *T2DM* | 21 (44.7)  39 (16.4) | 66 (42.6)  17 (21.8) | 46 (54.1)  26 (21.8) |
|  |  |  |  |  |
| **Insulin** (yes) | *T1DM*  *T2DM* | 46 (97.9)  92 (38.7) | 151 (98.1)  22 (28.2) | 84 (98.8)  47 (39.5) |
|  |  |  |  |  |
| **Treated primarily by a diabetologist** | *T1DM*  *T2DM* | 44 (93.6)  68 (28.6) | 139 (93.9)  16 (20.5) | 80 (95.2)  30 (25.2) |
|  |  |  |  |  |
| **Diabetes duration** (years) | *T1DM*  *T2DM* | 19.5 ± 10.8  13.0 ± 9.1 | 21.7 ± 13.8  11.0 ± 7.7 | 21.8 ± 14.8  12.9 ± 8.9 |
|  |  |  |  |  |
| **HbA_1c_-value** (%) |  |  |  |  |
| <6.5% | *T1DM*  *T2DM* | 3 (6.4)  61 (25.6) | 36 (23.2)  22 (28.2) | 22 (25.9)  29 (24.4) |
|  |  |  |  |  |
| 6.5 to<7.5% | *T1DM*  *T2DM* | 30 (63.8)  102 (42.9) | 69 (44.5)  28 (35.9) | 39 (45.9)  43 (36.1) |
|  |  |  |  |  |
| ≥ 7.5% | *T1DM*  *T2DM* | 12 (25.5)  44 (18.5) | 47 (30.3)  11 (14.1) | 17 (20.0)  30 (25.2) |
|  |  |  |  |  |
| unknown | *T1DM*  *T2DM* | 2 (4.3)  31 (13.0) | 3 (1.9)  17 (21.8) | 7 (8.2)  17 (14.3) |
| M=mean, SD= standard deviation, HbA_1c_= hemoglobin A1c | | | | |
